# Virtual reality 3D echocardiography in the assessment of tricuspid valve function after surgical closure of ventricular septal defect

**DOI:** 10.1186/1476-7120-5-8

**Published:** 2007-02-16

**Authors:** Goris Bol Raap, Anton HJ Koning, Thierry V Scohy, A Derk-Jan ten Harkel, Folkert J Meijboom, A Pieter Kappetein, Peter J van der Spek, Ad JJC Bogers

**Affiliations:** 1 Department of Cardiothoracic Surgery, Erasmus MC University Hospital, Rotterdam, The Netherlands; 2 Department of Bioinformatics, Erasmus MC University Hospital, Rotterdam, The Netherlands; 3 Department of Anesthesiology, Erasmus MC University Hospital, Rotterdam, The Netherlands; 4 Department of Pediatric Cardiology, Erasmus MC University Hospital, Rotterdam, The Netherlands; 5 Department of Cardiology, Erasmus MC University Hospital, Rotterdam, The Netherlands

## Abstract

**Background:**

This study was done to investigate the potential additional role of virtual reality, using three-dimensional (3D) echocardiographic holograms, in the postoperative assessment of tricuspid valve function after surgical closure of ventricular septal defect (VSD).

**Methods:**

12 data sets from intraoperative epicardial echocardiographic studies in 5 operations (patient age at operation 3 weeks to 4 years and bodyweight at operation 3.8 to 17.2 kg) after surgical closure of VSD were included in the study. The data sets were analysed as two-dimensional (2D) images on the screen of the ultrasound system as well as holograms in an I-space virtual reality (VR) system. The 2D images were assessed for tricuspid valve function. In the I-Space, a 6 degrees-of-freedom controller was used to create the necessary projectory positions and cutting planes in the hologram. The holograms were used for additional assessment of tricuspid valve leaflet mobility.

**Results:**

All data sets could be used for 2D as well as holographic analysis. In all data sets the area of interest could be identified. The 2D analysis showed no tricuspid valve stenosis or regurgitation. Leaflet mobility was considered normal. In the virtual reality of the I-Space, all data sets allowed to assess the tricuspid leaflet level in a single holographic representation. In 3 holograms the septal leaflet showed restricted mobility that was not appreciated in the 2D echocardiogram. In 4 data sets the posterior leaflet and the tricuspid papillary apparatus were not completely included.

**Conclusion:**

This report shows that dynamic holographic imaging of intraoperative postoperative echocardiographic data regarding tricuspid valve function after VSD closure is feasible. Holographic analysis allows for additional tricuspid valve leaflet mobility analysis. The large size of the probe, in relation to small size of the patient, may preclude a complete data set. At the moment the requirement of an I-Space VR system limits the applicability in virtual reality 3D echocardiography in clinical practice.

## Background

As recently described the mental conceptualization and evaluation of the intracardiac anatomy from multiple two-dimensional (2D) echocardiographic images, is complicated by cardiac dynamics [1-4]. Three-dimensional (3D) echocardiography facilitates simplification of this process by offering a direct representation of the cardiac anatomy throughout the cardiac cycle [4]. To appreciate the full 3D potential of these datasets, virtual reality technology can be applied and used for further interpretation of the data of cardiac echocardiography [4-9]. In this regard we applied holographic analysis of intraoperative epicardial echocardiography in order to further contribute to the discussion of tricuspid valve function after surgical closure of ventricular septal defect (VSD) [10-13].

## Methods

### Patients and data sets

12 data sets from intraoperative epicardial echocardiographic studies in 5 operations (patient age at operation 3, 6, 7 weeks, 9 months and 4 years and bodyweight at operation 3.8, 3.9, 4.5, 5.2 and 17.2 kg.) were included in the study. Epicardial echo was indicated in these patients on clinical grounds to obtain additional 2D imaging on top of the pre-operative echo assessment. During the epicardial echocardiography, the 3D data sets were obtained as well.

All operations concerned closure of VSD, in 1/5 as isolated defect, in 3/5 in the setting of correction of tetralogy of Fallot, in 1/5 in combination with a double-chambered right ventricle. In none of the operations the technique of temporary tricuspid detachment was used. In 3 of the operations sutures of the VSD patch were anchored in the base of the tricuspid valve annulus. The postcorrection epicardial echocardiography was performed as previously described [14].

### Three-dimensional echocardiographic data acquisition

In the setting described previously [4] the data sets were acquired with the iE33 ultrasound system equipped with 3D data acquisition software (Philips Medical Systems, Andover, MA, USA), using an X3-1 broadband matrix array transducer (Philips Medical Systems, Andover, MA, USA) that was used epicardially after the surgical correction. Epicardial echocardiography took 4–5 minutes in each patient and included 2D echo analysis and acquisition of the 3D data sets. The echocardiographic study, including real-time 3D acquisition, was done with ECG gating.

Data processing consisted of the creation of a Cartesian volume in DICOM 3.0 format by interpolating the original data using the export module of the Q-lab ultrasound data analysis application (Philips Medical Systems, Andover, MA, USA), using the highest quality settings.

The 3D image data were transferred to an SGI Prism computer (Silicon Graphics, Mountain View, CA, USA) driving the I-space virtual reality (VR) system. A simple format conversion was required to be able to load the data in the I-Space software. In total the processing and the conversion only took 1–2 minutes per dataset. Holographic analysis took 15–18 minutes for a single data set

### Visualisation in the virtual reality environment

The BARCO I-space (Barco, Kortrijk, Belgium) installed at the Erasmus MC (Erasmus MC, Rotterdam, The Netherlands) is a so-called four-walled CAVE™-like VR system [15]. In the I-Space researchers are surrounded by computer-generated stereo images, which are projected by 4 high quality DLP-projectors on three walls and the floor of the projection room [see Additional file 1]. The CAVORE volume rendering application [16] is used to investigate 3D ultrasound images during the cardiac cycle [10]. In the I-Space, this results in an animated hologram of the dataset being visualised, floating in space in front of the viewers. The viewers wear a pair of lightweight glasses with polarising lenses that allows seeing the hologram with depth [see Additional files 1]. A wireless 6 degrees-of-freedom (DOF) controller which emits a virtual pointer is used for manipulation of and interaction with this hologram. A cutting plane attached to the pointer allows assessment of the interior of the heart. A transfer function widget operated by the pointer controls the contrast and transparency of the rendered image. The cardiothoracic surgeon performed the virtual reality 3D echo analysis.

## Results

In the 2D representation of the datasets the area of interest could adequately be visualized in all of the 12 data sets. The data sets were used intraoperatively for clinical assessment of the result of the operation. In none of the data sets 2D analysis showed tricuspid stenosis or regurgitation. Mobility of the anterior and septal tricuspid valve leaflets was considered to be normal in all data sets. In 4 data sets, the posterior leaflet was not included, because of the limitations of epicardial access of a large probe in a small patient. In the other 8 data sets no abnormalities of posterior leaflet motion were noted.

All the 3D datasets could be adequately analyzed in the I-Space. Virtual reality of the datasets allowed a complete assessment of the area of interest and easy investigation of the area of the reconstruction. The holograms of the area of closure of the VSD were adequately visualised from the right side of the ventricular septum.

Assessment of the tricuspid valve leaflet motion could well be accomplished from a right atrial view. In 3 data sets (in 3 different patients) a restriction of the mobility of the septal leaflet was noted, that was not appreciated in the 2D analyses. In all these 3 patients the VSD patch was anchored in the base of the septal leaflet of the tricuspid valve. No abnormalities in anterior leaflet motion were noted. In the limited nearfield of the epicardial echocardiography only 8 posterior leaflets could be assessed, their mobility was considered normal. The holograms confirmed that the papillary apparatus in these 4 data sets was incompletely acquired.

## Discussion

A virtual reality approach is presented to analyse the results of surgery for congenital VSD. The 3D echocardiographic data sets acquired epicardially and generated by a commercially available echo system were used to construct a dynamic hologram inside an I-Space. All the datasets could be adequately analyzed in the I-Space, in which a single 3D dataset is sufficient to create every view of interest.

Currently, large-scale VR facilities like the I-Space are only available in a limited number of research centres throughout the world. Desktop applications are being developed, for instance the Personal Space Station PSS™ (Personal Space Technologies, Amsterdam, The Netherlands). Therefore, the representation and analysis of diagnostic 3D echocardiography by virtual reality has only been described very recently [4]. As a new application, this experience is extended to postoperative control of the results of surgery for congenital VSD, in datasets derived epicardially. This is a relevant topic because in closure of a VSD, the sutures of the patch are often anchored in the base of the septal and anterior tricuspid valve leaflet and sometimes the tricuspid valve may be temporarily detached in the area of the septal and anterior leaflets in order to optimise the surgical approach [10-12]. Until now, tricuspid valve function was shown not to be affected by these techniques [10,11]. Recently, temporary tricuspid detachment was even advocated to avoid early postoperative tricuspid valve function due to inadvertent traction [12]. As a consequence tricuspid valve function and growth are matters of interest in this regard.

However, because the area of interest is in the nearfield of the echoprobe, a complete data set may be precluded, especially because of the large size of the probe that was available at the time of the study in relation to the small size of the patients and the limited exposure of the heart in them. Although no tricuspid valve stenosis or regurgitation was found in our series by 2D analysis of the data sets, in 3 of them a restricted mobility of the septal leaflet was found by holographic analysis. This suggests that holographic analysis may provide additional data beyond conventional analysis. In general, differences in interpretation between 2D and 3D echo can well be explained [17]. In 2D echocardiography, tricuspid valve analysis only allows analysis of two leaflets per image plane. Usually these are the anterior and septal leaflets, or the anterior and posterior leaflets. Hardly ever the posterior and septal leaflets can be visualized in one image plane and never all three leaflets. In contrast, 3D echocardiography allows all three leaflets to be seen in one view.

The virtual reality analysis can be done by the anatomy-expert, for instance the cardiothoracic surgeon performing the operation, and depends to a lesser extent on the echocardiography-expert, for instance the cardiologist making the diagnosis.

Virtual reality in this regard provides an additional resource for postoperative quality control as well as for education with regard to the intracardiac repair of congenital VSD.

With the I-Space technology the complex postoperative cardiac anatomy of the closed congenital VSD, in relation to tricuspid valve function, can be appropriately visualised in virtual reality. Unfortunately, at present the colour-Doppler data cannot yet be transferred to the I-Space, as the data is only available in a proprietary format.

For the clinical practice, virtual reality 3D echocardiography should be implemented on smaller systems, like desktop displays or single screen projection systems, to allow bedside use or application in the operating theatre or conference room.

In conclusion, epicardially derived data sets may be incomplete for nearfield structures, especially when probe size mismatches with patient size. Still, the resulting 3D data sets nicely provide holographic data, which may provide additional information on tricuspid valve function after VSD closure

## Competing interests

The author(s) declare that they have no competing interests.

## Authors' contributions

GBR conceived the study, participated in analysis of the data and drafted the manuscript. AHJK imported the 3D datasets into the virtual reality system, participated analysis of the data and contributed to the drafting of the manuscript. TS participated in acquisition and analysis of the data and participated in revising the manuscript. DJH, FJM, APK and PJS revised the manuscript with important intellectual content. AJJCB conceived the study, participated in analysis of the data, revised the manuscript with important intellectual content and gave final approval of the version to be published. All authors read and approved the final manuscript.

**Figure 1:**
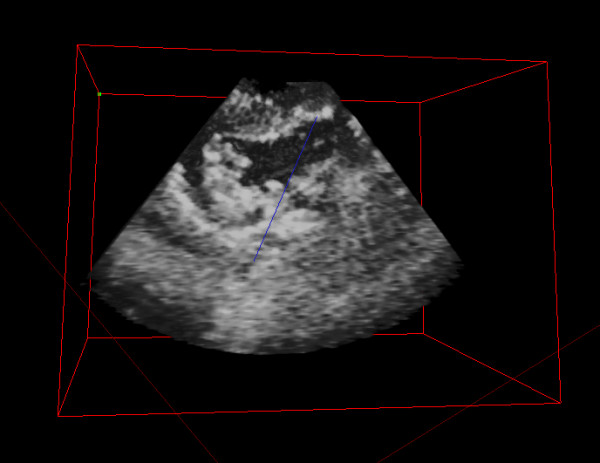
The photo shows the 3D hologram of the ventricular septal defect repaired with a patch.

**Figure 2:**
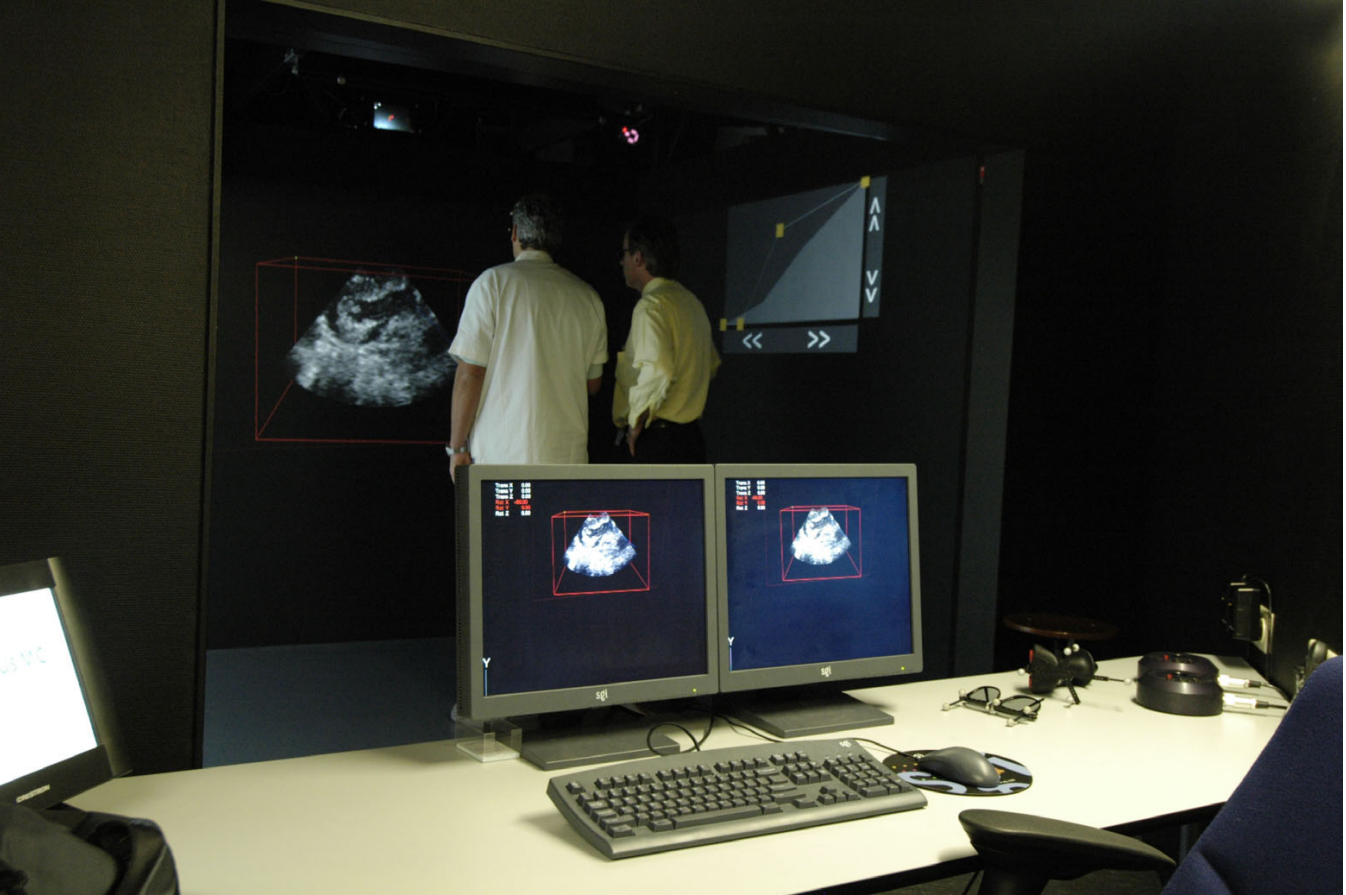
Image showing the design of the I-space.

## Supplementary Material

Additional file 1 Design of the I-space and interpretation by two experts of the 3D hologram of a right atrial view of the repaired ventricular septal defect with a patch. Click here for file
